# Nutraceutical Potential of Anthocyanins: A Comprehensive Treatise

**DOI:** 10.1002/fsn3.70164

**Published:** 2025-05-04

**Authors:** Ahmad Mujtaba Noman, Muhammad Tauseef Sultan, Muhammad Maaz, Aimen Mazhar, Naima Tariq, Muhammad Imran, Muzzamal Hussain, Ahmed Mujtaba, Mohamed A. Abdelgawad, Ehab M. Mostafa, Mohammed M. Ghoneim, Samy Selim, Entessar Al Jbawi

**Affiliations:** ^1^ Department of Human Nutrition, Faculty of Food Science and Nutrition Bahauddin Zakariya University Multan Pakistan; ^2^ TIMES Institute Multan Pakistan; ^3^ Departmnet of Food Science and Technology, Faculty of Food Science and Nutrition Bahauddin Zakariya University Multan Pakistan; ^4^ Department of Food Science and Technology University of Narowal Narowal Pakistan; ^5^ Department of Food Science Government College University Faisalabad Faisalabad Pakistan; ^6^ Department of Food Sciences and Technology, Faculty of Engineering Sciences and Technology Hamdard University Islamabad Islamabad Pakistan; ^7^ Department of Pharmaceutical Chemistry, College of Pharmacy Jouf University Sakaka Saudi Arabia; ^8^ Department of Pharmacognosy, College of Pharmacy Jouf University Sakaka Saudi Arabia; ^9^ Department of Pharmacy Practice, College of Pharmacy AlMaarefa University Riyadh Saudi Arabia; ^10^ Department of Clinical Laboratory Sciences, College of Applied Medical Sciences Jouf University Sakaka Saudi Arabia; ^11^ Agricultural Extension Directorate, MAAR Damascus Syria

**Keywords:** Anthocyanins, anticancer, antidiabetic, gut‐brain axis, nutraceutical applications, pharmacokinetics

## Abstract

Anthocyanins (Anthos; flower and kyanos; blue) are natural coloring compounds from the flavonoids class that include cyanidin, peonidin, delphinidin, malvidin, pelargonidin, and petunidin. Recently, the role of anthocyanins in disease prevention, especially inflammation, diabetes, cancer, neuro‐disorders, hepato‐renal protective, and immuno‐modulation properties has been highlighted. The current review covered the literature on the pharmacokinetics and pharmacological effects of anthocyanins, especially absorption, distribution, metabolism, and excretion (ADME). The discussion on molecular mechanisms underlying their therapeutic effects is the limelight of the article. The GLUT1, GLUT3, SGLT1, SMCT1, and SMCT2 are the main carriers involved in the transportation of anthocyanins in the gastrointestinal tract. The anthocyanins exert their anticancer effects by reducing the expression of IL‐6, IL‐1β, TNF‐β, COX‐2, downregulation of NF‐kB, *EZH2, MDR1, Akt*, and modulation of P13K/AKT and AMPK/mTOR pathways. The reduction in α‐amylase and α‐glucosidase and improved FFAR1 activity results in antidiabetic effects. The regulation of PGC‐1α/NRF2/TFAM, p‐PI3K/Akt/GSK3β, and Nrf2/HO‐1 prevents neurodegeneration. The anthocyanins impose hepato‐renal protective effects via ameliorating NLRP3 inflammasome, inhibiting MDA, GSSG, iNOS, HO‐1, ICAM‐1, β2‐microglobulin, and MPO activity, and improved SOD, CAT, and GSH activity. Anthocyanins promote beneficial gut microbiota and enhance SCFA production, thus inhibiting pro‐inflammatory markers. The immuno‐modulatory impact of anthocyanins involves the reduction of CRP, P‐selectin, C1q, and C4. Anthocyanins reduce LDL, VLDL, TGs, and TC via improved GBA and upregulation of ATP6 V0C, ZO‐1, and ATG4D expression. The WHO/FAO suggested that 2.5 mg/kg/day of grape‐skin extracts of anthocyanins are safe, and China recommended that 50 mg/day of anthocyanins are safe for consumption. In a nutshell, the multifaceted health benefits of anthocyanins make them promising candidates for disease prevention and therapeutic interventions.

## Graphical abstract

Anthocyanins reduce LDL, VLDL, TGs, and TC via improved GBA and upregulation of ATP6 V0C, ZO‐1, and ATG4D expression. The WHO/FAO suggested that 2.5 mg/kg/day of grape‐skin extracts of anthocyanins is safe, and China recommended that 50 mg/day of anthocyanins is safe for consumption. The current review focuses on anthocyanin sources, stability, pharmacokinetics, pharmacological aspects, safety, and applications.

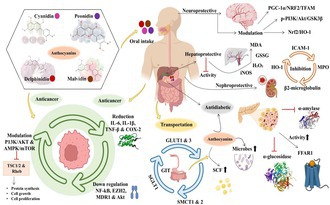

**Abbreviations**
Akt: protein kinase B
AMPK: adenosine monophosphate‐activated protein kinase
Bcl‐2: B‐cell lymphoma 2
C3G: cyanidin‐3‐glucoside
CAT: catalase
COX‐2: cyclooxygenase 2
Cy3glc: cyanidin‐3‐O‐β‐glucopyranoside
DPPH: 2,2‐diphenyl‐1‐picrylhydrazyl
EZH2: enhancer of zeste homolog 2
FFAR1: free fatty acid receptor 1
FRAP: ferric reducing antioxidant power
GLUT1: glucose transporter 1
GLUT3: glucose transporter 3
GSK3β: glycogen synthase kinase‐3 beta
GSSG: glutathione disulfide
H₂O₂: hydrogen peroxide
HO‐1: heme oxygenase‐1
HPLC: high‐performance liquid chromatography
ICAM‐1: intercellular adhesion molecule 1
IL‐1β: interleukin 1 beta
IL‐6: interleukin 6
INF‐γ: interferon‐gamma
iNOS: Inducible nitric oxide synthase
JNK: c‐Jun N‐terminal kinase
M3G: malvidin‐3‐O‐galactoside
MDA: malondialdehyde
MDR1: multidrug resistance protein 1
MPO: myeloperoxidase
mTOR: mammalian target of rapamycin
NF‐kB: nuclear factor‐kappa B
NRF2: nuclear factor erythroid 2–related factor 2
OS: oxidative stress
PGC‐1α: peroxisome proliferator‐activated receptor‐γ coactivator 1α
PGE2: prostaglandin E2
PPAR‐γ: peroxisome proliferator‐activated receptor gamma
PTEN: phosphatase and tensin homolog
RONS: reactive oxygen and nitrogen species
SDH: succinate dehydrogenase
SGLT1: sodium‐glucose cotransporter 1
SMCT1: sodium‐coupled monocarboxylate transporter 1
SMCT2: sodium‐coupled monocarboxylate transporter 2
SOD: superoxide dismutase
TFAM: mitochondrial transcription factor A
TLR4: toll‐like receptor 4
TNF‐β: tumor necrosis factor beta
TSGs: tumor suppressor genes

## 1 Introduction

The bioactive compounds have significantly enhanced life expectancy by improving life quality and lifespan. Phytochemicals with remarkable therapeutic potential are practical approaches to managing health challenges. The availability and affordability of bioactive compounds have made them preferable for local folks, herbalists, and nutraceutical and pharmaceutical industries (Ahad et al. 2021). These bioactive compounds have been widely used to treat acute conditions (fever, headache, flu) as well as chronic metabolic syndromes, such as diabetes mellitus (DM), hypertension (HTN), cardiovascular diseases (CVDs), cancer, neurodegenerative disorders, gastrointestinal (GIT) issues, pulmonary abnormalities, and hepato‐renal ailments (Jiang 2019).

**FIGURE 1:**
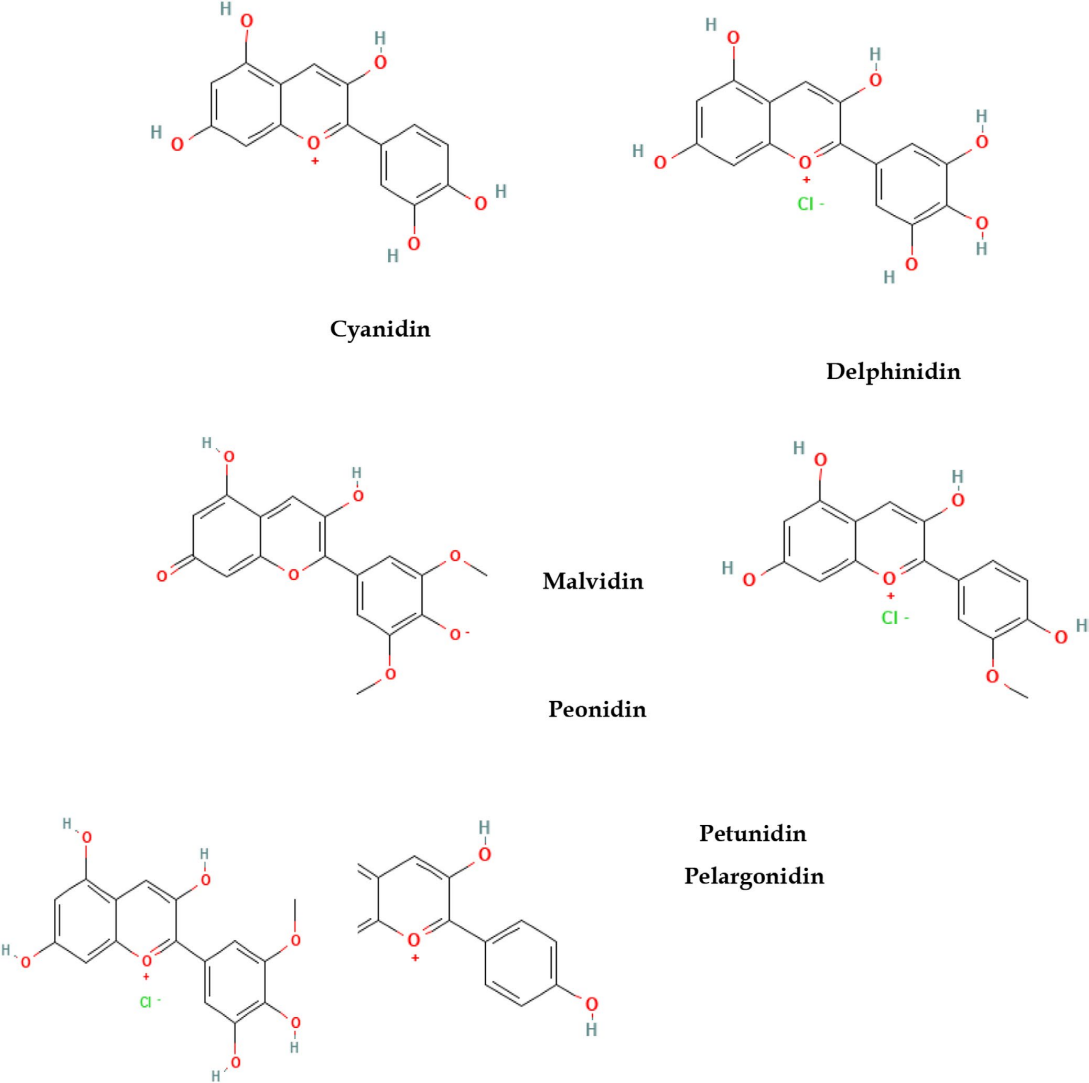
Chemical structures of major anthocyanins.

Plant‐based foods such as fruits and vegetables are rich sources of numerous bioactive compounds. From providing eye‐appealing colors to pharmacological potentials, these bioactive compounds have been acknowledged in industries and healthcare systems. Among these, anthocyanins are water‐soluble coloring pigments belonging to flavonoids, abundantly present in blue, red, and purple fruits, vegetables, and flowers. Anthocyanins have gained attention as natural colorants for functional foods and beverages like marmalades, yogurts, juices, and bakery products (Bendokas, Skemiene, et al. 2020). Anthocyanins are sensitive to temperature, light, pH, and other processing factors; thus, their stability is a significant limitation and hinders their commercial and pharmaceutical applications (Xue et al. 2024). Figure 1 shows chemical structures of major anthocyanins.

The bioactivities of anthocyanins are highly dependent on their structure, although all forms, extracted from different sources with different compositions and amounts of anthocyanins, are biologically active. Studies have proved their role as antioxidants (Garcia and Blesso 2021), anti‐inflammatory agents (Merecz‐Sadowska et al. 2023), anticancer agents (Kowalczyk et al. 2024), antidiabetes agents (Les et al. 2021), hypolipidemic agents (Herrera‐Balandrano et al. 2021), neuroprotective agents (Zhong et al. 2023), gut health improvement agents (Song, Shen, Zhou et al. 2021), hepato‐renal protection agents (Orororo and Asagba 2022), and antimicrobial agents (Ha and Le 2022). The significance of undertaking this thorough review lies in delivering a current and detailed analysis of anthocyanins' therapeutic potential. This review aims to highlight diverse therapeutic properties and uncover any discoveries that have surfaced in recent years.

## 2 Methodology

For the current review, data was collected from different search engines such as Google Scholar, PubMed, Science Direct, and Scopus. Moreover, papers from well‐known publishers with high citations were selected. The current review covers the most recent studies (2015–2024). However, a few studies concerning some clinical trials are old.

### 2.1 Sources, and Consumption of Anthocyanins

Anthocyanins are primarily present in colorful fruits, vegetables, and flowers, providing them with blue, purple, red, and black colors. However, quantity and composition can vary depending on plant genotype, climatic circumstances, and fruit/vegetable maturity. Thus, fruits and vegetables in the human diet are significant sources of anthocyanins, and they can abundantly exist in the flesh and peel with different content and composition (Tena et al. 2020). Recently, ~600 anthocyanins have been identified; among these, cyanidin, peonidin, delphinidin, malvidin, pelargonidin, and petunidin are plenteous. Berries, grapes, currants, and some tropical fruits are rich sources of anthocyanins, while vegetables include leafy vegetables, tubers, grains, and roots (de Morais et al. 2020). Stanys et al. (2019) identified cyanidins as the most dominant anthocyanins in *Ribes nigrum* (Blackcurrant) species. Previously, Veberic et al. (2015) found that delphinidins are the main anthocyanins, making up over 57.6%, whereas cyanidins are second with 23.7% content and malvidins reaching ~14.1% of the total in bilberries. Furthermore, they stated that delphinidins range from 66.7% to 70.2% in blackcurrant. However, Bendokas, Stanys, et al. (2020) reported that cyanidins are the most prominent anthocyanins in sweet cherry, elderberry, sour cherry, and redcurrant. Malvidins are the key anthocyanin in grapes, ranging from 35.8% to 67.1%. Similarly, cyanidins are the main anthocyanins in vegetables, but only eggplant contains delphinidins as a major constituent (Blackhall et al. 2018). Red chicory has been shown to have the maximum anthocyanin concentration in vegetables, though it is 2–20 fold less than berries (Oladzadabbasabadi et al. 2022). In the US, the consumption of anthocyanins fluctuates from 9 mg/day, while in Europe, it is 19 mg/day but reaches 28 mg in some European communities. The primary sources of anthocyanins are berries (39% in the US and 43% in Europe) and fruits (9% in the US and 19% in Europe) (Kim et al. 2016; Vogiatzoglou et al. 2015). The data regarding sources, concentration, and type of anthocyanins in different fruits and vegetables is presented in Table 1.

**TABLE 1:** Sources, concentration, and type of anthocyanins in different fruits and vegetables.

Source	Concentration mg 100 g − 1 FW	Major anthocyanins	Reference
Bilberry	771.5	Dp3gal, Cy3glc, Cy3gal, Mv3glc, Cy3ara, Dp3glc	Veberic et al. (2015)
Golden currant	614.6	Pn3rut, Cy3rut, Cy3glc	Stanys et al. (2019)
Elderberry	570.0	Cy3sam, Cy3glc	Mikulic‐Petkovsek et al. (2014)
Blackcurrant	477.4	Dp3rut, Dp3glc, Cy3glc	Veberic et al. (2015)
Sweet cherry	245.0	Cy3rut, Pn3rut	Blackhall et al. (2018)
Grapes	116.3	Mv3glc, Pn3glc, Dp3glc, Pt3glc	Dimitrovska et al. (2011)
Sour cherry	146.1	Cy3rut	Bendokas et al. (2013)
Black carrot	125.4	Cy3xylglcgal, Cy3xylgal	Algarra et al. (2014)
Red cabbage	23.6	Cy3glc, Cy3diglc5glc, Dp3rut	Tong et al. (2017)
Eggplant	8.8	Dp3glc, Dp3rut	Frond et al. (2019)

### 2.2 Stability of Anthocyanins

The stability of anthocyanins is critical due to their sensitivity toward pH, light, heat, oxygen, and other processing conditions. Studies have proved that anthocyanins are more stable under acidic circumstances; at seven pH, they are usually degraded. Furthermore, at different pH, ions of anthocyanins depicted their effect and generated pigments of various colors. For instance, at a pH of 1.0, flavylium is the prominent species and provides purple and red pigments, whereas at pH 2.0–4.0, the blue quinoidal species dominates (Enaru et al. 2021). Besides pH, temperature and storage duration also crucially affect anthocyanin concentrations. Almost 11% of rosella anthocyanins were lost at 4°C (storage for 60 days), while ~99% of anthocyanins were degraded when stored at 37°C for the same duration (Sinela et al. 2017). Anthocyanins of redcurrant berry extract are more stable in light and room temperature than golden currant and gooseberry extracts. However, under cold conditions (4°C) for 83 days, 90% of redcurrant, ~80% of gooseberry, and 50% of blackcurrant anthocyanins remained undamaged (Bendokas et al. 2013).

Thermal stability could be the biggest challenge to anthocyanins' stability, and most anthocyanins and their nutritional value are lost during beverage production. Thermal processing contributes to the loss of 35% anthocyanins, and even short‐term thermal treatment for 5 s (85°C) outcomes in a loss of 9% strawberry juice anthocyanins, and pasteurization for 15 min caused a loss of 21% anthocyanins (Weber and Larsen 2017). Approximately 41.2% of anthocyanins were lost in boiling red cabbage while steaming or stir‐frying did not affect anthocyanin concentration. The hydrophilic nature of anthocyanins probably results in their loss by leaching in boiling due to high water solubility (Murador et al. 2016). New techniques are being developed to limit the loss of anthocyanins; yeast mannoproteins at pH 7.0 are being applied, and the complexes have been found to prevent anthocyanins' thermal degradation at 80°C–126°C. The addition of acerola juice with montmorillonite protected more than 50% of anthocyanins, irrespective of time or pH fluctuations (Ribeiro et al. 2018).

Novel isolation methods and other strategies are adopted to enhance anthocyanin stability. Co‐pigmentation is another effective tool that can strengthen anthocyanin stability. Phenolic acids (hydroxycinnamic and hydroxybenzoic acids) are more suitable and studied pigments, which can improve anthocyanin stability. Babaloo and Jamei (2018) reported that caffeic acid proves more effective in providing stability than benzoic and coumaric acids. Encapsulation with polysaccharides (β‐cyclodextrin, maltodextrin, Arabic gum) is vital for stabilization. The protective impact of β‐cyclodextrin was proved for blackberry anthocyanins after thermal treatment at 90°C for 2 h (Fernandes et al. 2018). The block freeze concentration technique has been applied to extract anthocyanins from strawberries. The foam mat drying method is another effective dehydration practice to produce powder from pulp, and only a 7%–9% reduction of anthocyanins was detected after the storage of jambolana juice powder for 150 days. Additionally, ~80%–100% of anthocyanins remained intact after foam‐mat freeze‐drying to produce powder from blueberry juice, and the most stable anthocyanin was Cy3glc (Darniadi et al. 2019; Jaster et al. 2018).

### 2.3 Pharmacokinetics and Bioavailability

Pharmacokinetics determines the therapeutic outcomes of specific bioactive compounds, and it depends on the concentration and availability of the compound from the source and its bioavailability in the host (absorption in the gut). Moreover, chemical structure, interaction with the food matrix, existence of other dietary compounds, and individual genomics and physiology are other crucial factors influencing pharmacokinetics and health benefits. Regarding anthocyanins, glycosylated anthocyanins with sugar moiety are more resilient to digestion than aglycones and mono‐glucosides. Moreover, acylated anthocyanins have lesser digestibility than non‐acylated due to decreased polarity and interruption of acyl groups (Yang et al. 2018). The stomach is the first place of digestion for anthocyanins, and anthocyanins' chemical structure and molecular weight define the digestion and absorption rate. A minor quantity of anthocyanins in the stomach crosses the gastric cell barrier and is rapidly detected in plasma. Anthocyanins remain stable in the stomach, and 75%–79% of ingested anthocyanins are recoverable from the leftovers during rat studies, and 75%–88% are recoverable during in vitro trials (Han et al. 2019).

High glucose substantially decreased the gastric cell barrier, resulting in affected anthocyanins gastric absorption. The human gastric cell (MKN‐28) model verified that M3G had a weak interaction with the transport receptor (XylE), showing a higher absorption rate than mono‐glycosides. In contrast, due to the huge molecular size, pyranoanthocyanins had half the transport efficiency compared to M3G (Oliveira et al. 2016). Gastric bilitranslocase, a membrane protein transporter that transfers anthocyanins across the gastric barrier, and the anthocyanins‐glycosides moiety meet the structural condition given by bilitranslocase, thus being absorbed without de‐glycosylation. Further, glucose transporters (GLUT1 and GLUT3) are also involved in the gastric uptake of anthocyanins (Kalt 2019). Some other transporters, like SMCT1 and SMCT2, and organic cation/anion (OCT1 and OAT2) are also involved in the gastric absorption of anthocyanins (Manolescu et al. 2019). Previously, it was considered that the stomach is the only site for the digestion and absorption of anthocyanins, but the discovery of catechol‐O‐methyl transferase, sulfotransferase, and UDP‐glucuronosyltransferase in stomach epithelium makes it clear that the stomach is also involved in metabolism (Han et al. 2019). Concerning this, Han et al. (2020) investigated the absorption of red wine anthocyanins in Caco‐2 cells and found M3G during in vitro digestion, evidencing gastric metabolism.

The jejunum is the central location for anthocyanin absorption, and ~5% of total anthocyanins were absorbed across the small intestine epithelium. The anthocyanin absorption in the small intestine occurs in two ways: either through passive diffusion or active transport via membrane carriers. Like gastric absorption, undamaged glycosylated anthocyanins are absorbed in intestinal epithelial cells by SGLT1 and GLUT2 (Zia Ul Haq et al. 2016). An OATP transporter has recently been identified, contributing to the β‐type acylated anthocyanin absorption across rat jejunum epithelium. However, its absorption was low, ranging from 0.2%–2.2% (Hahm et al. 2021). In contrast, passive diffusion involves hydrolyzation of anthocyanins via border enzymes (β‐galactosidase, α‐rhamnosidase and β‐glucuronidase) to their aglycone. Recent use of the Caco‐2 cells model for evaluating anthocyanin transport proposed that the effectiveness of transport depends on the type of aglycone and attached sugar, whether the anthocyanin is polymeric or not (Kamiloglu et al. 2015). Similarly, the presence of hydroxyl groups can also impact the transportation of anthocyanin. Pacheco‐Palencia et al. (2010) suggested that blueberry‐derived delphinidin‐3‐glucoside had less transport proficiency due to more hydroxyl group numbers on delphinidin than malvidin and peonidin glucosides. In addition, the Caco‐2 cell model verified that cyanidin glucoside had a greater transport rate rather than its galactoside form. However, cyanidin‐3‐rutinoside and C3G elucidated similar absorption rates, indicating that sugar slightly affects anthocyanin absorption.

After absorption into enterocytes, anthocyanins are distributed to the liver through the bloodstream for additional reactions, enhancing their solubility in water and, finally, their elimination in the urine. However, the unabsorbed anthocyanins experience bio‐transformation through bacterial catabolism, enhancing their bioavailability and permitting absorption through the colonic wall. *Bacteroides* spp., *E. casseliflavus* , and *Eubacterium* spp. are major bacterial strains that produce various enzymes like β‐glucosidase and α‐rhamnosidase for microbial breakdown (Rowland et al. 2018). Anthocyanins are hydrolyzed in the colon by microbial enzymes, having the ability to cleave sugar linkages to release anthocyanin aglycones, but these aglycones are unstable and quickly converted into phloroglucinol aldehydes (Pas) (Victoria‐Campos et al. 2022). The M3G is totally metabolized to syringic acid, and peonidin is transformed into vanillic acid (Makarewicz et al. 2021); likewise, pelargonidin‐3‐O‐glucoside breaks into 4‐hydroxybenzoic acid and petunidin‐based anthocyanins finally produce gallic acid by O‐demethylation (Fernandes et al. 2014). It has been suggested that anthocyanin‐derived metabolites validate more stability and bioactivity than parent anthocyanins and improve the human gut microbial population. However, colonic microbes are influenced by factors such as dietary patterns, age, host genetics, weight, and antibiotic treatment, and they vary among people. Subsequently, this variation also affects anthocyanin bioavailability and metabolism.

### 2.4 Antioxidant Potential

Overproduction or imbalance between RONS, which are highly unstable, leads to OS/inflammation, DNA damage, genetic mutation, and other chronic metabolic disorders. However, bioactive compounds have the potential to scavenge RONS, thus reducing inflammation and the risk of chronic ailments. Numerous studies have proved the antioxidant capacity of anthocyanins; however, the source and nature of anthocyanin can affect the antioxidant capacity. Generally, the antioxidant activity of anthocyanins can be explained in two mechanisms: the Hydrogen atom donator (HAT) and the single‐electron transfer (SET). The HAT mechanism involves the removal of hydrogen atoms from antioxidants (AH+) by free radical R•, which stabilize the free radical. Meanwhile, the SET mechanism includes the donation of an electron to the free radical by the antioxidant (AH+), thus allowing free radicals to convert into a stable form (Foroutani et al. 2024). Using eutectic solvents, Zannou and Koca (2022) determined the blackberry anthocyanins antioxidant activity. They found significant quantities of cyanidin chloride, C3G, pelargonidin‐3‐glucoside, and cyanidin‐3‐rutinoside. Moreover, blackberry exhibited high TPC (9.35 ± 0.39 mg GAE/G), anthocyanin content (115.37 ± 0.43), metal chelating activity (4771.25 ± 83.58 mg TE/100 g), and reducing power (83.08 ± 3.78).

Studies on different environmental factors have proved that temperature variations could affect antioxidant activity and alter the anthocyanin content. Sudheeran et al. (2018) studied the effects of temperature on red and green mango by storing them for 3 weeks at 5°C, 8°C, or 12°C. They concluded that anthocyanins and flavonoid content increased 2 times in red mango, and red mango proved more resistant to temperature stress with non‐significant changes in physiological parameters. Zhou et al. (2020) investigated the impact of blueberry anthocyanins on gut microflora and determined anthocyanins/antioxidant activity on blueberry. They concluded that M3G was the key anthocyanin, tailed by malvidin‐3‐O‐galactoside and petunidin‐3‐O‐glucoside. They accounted for ~44.81% of total anthocyanins in blueberry extract. The ABTS + showed a lower value than DPPH (14.99 μg/mL) and FRAP (26.48 μg/mL). Moreover, the study findings suggested that blueberry anthocyanins augmented the number of *Bifidobacterium* spp.

The extraction method could be another limitation, substantially affecting the antioxidant potential of anthocyanins. In this context, Li, Chen, et al. (2021) examined the impact of different extraction techniques on the antioxidant capacity of blueberry anthocyanins. They concluded that solvent extraction proved more suitable than ultrasonication and enzyme extraction methods. Kumari et al. (2022) determined the anthocyanin profile and antioxidant potential of Indian rose varieties by using HPLC. They found that the Ashwini variety exhibited the highest TPC (427.59 ± 3.47 mg GAE/100 g), FRAP (397.15 ± 0.82 μmol trolox/g), and DPPH activity (93.47% ± 0.19%). Furthermore, two dominant anthocyanins were cyanidin 3,5‐di‐O‐glucoside and pelargonidin 3,5‐di‐O‐glucoside, and the first one is prominent in red/pink colored varieties, while the second is found in the orange variety. The cyanidin 3,5‐di‐O‐glucoside content was (497.79 mg/100 g) in Ashwini, and pelargonidin 3,5‐di‐O‐glucoside concentration was (185.43 mg/100 g) in Suryakiran.

### 2.5 Anti‐Inflammatory and Anticancer Activity

Cancer is one of the leading causes of morbidities and fatalities worldwide, covering various factors in its progression. Inflammation and OS are significant factors contributing to cancer prevalence. The underlying pathophysiology of cancer is a complex process involving multiple pathways and mediators. The primary cause of inflammation is an injury at the molecular level due to pathogenic infection, chemicals, toxins, heavy metals, radiation, food components, and over‐the‐counter (OTC) drugs. These substances have been reported to alter molecular mechanisms, resulting in over‐production or imbalance between RONS, OS, genetic mutations, uncontrolled cell proliferation, metastasis, and oncogenesis (Singh et al. 2019). The pro‐inflammatory biomarkers (IL‐1, IL‐6, TNF‐α) promote the inflammatory process and mutate proto‐oncogenes (*RAS, ERK, Wnt, TRK, CMYC, fos, K‐ras, β‐catenin*), converting them into oncogenes (*HER2, EML4‐ALK, BCR/ABL1*), thus inducing cell proliferation. Moreover, the downregulation of TSGs (*p53, pRb, APC, PTEN*), antioxidant enzymes (SOD, GSH, CAT, GPx) suppression, and triggering of cancer pathways/axis (PI3K/AKT/mTOR, Ras/MAPK, GSK3, TGFβ signaling) are also remarkably contributing to cancer development (Chhichholiya et al. 2024).

Several in vitro and in vivo studies of anthocyanins have proved their anti‐inflammatory and anticancer potential against various cancers. The anthocyanin supplementation (320 mg/day) for 4 weeks significantly reduced IL‐6 production and TNF‐α and COX‐2 expression, downregulated NF‐kB dependent gene, and upregulated *PPAR‐γ* gene expression (Aboonabi and Aboonabi 2020). The AMP‐activated protein kinase (AMPK) adjusts energy levels and is a key target in cancer prevention and treatment. Tsai et al. (2023) investigated the anticancer activity of *hibiscus* anthocyanins extracts against colorectal cancer (CRC) cell lines through AMPK pathways. They concluded that (0, 1, 2, and 3 mg/mL) anthocyanins induced apoptosis in LoVo cell lines via Fas‐mediated protein activation, upregulation of caspase‐8/tBid, and improved caspase‐3. Numerous studies have clearly discussed the association between growth factors and neoplasm. Epidermal growth factor receptor (EGFR) is a tyrosine kinase receptor that plays a significant role in CRC by stimulating several pathways like RAF, MAPK, RAS, and MEK. EGF receptor inhibition has been reported as a practical approach to treating CRC (Janani et al. 2022). Moreover, the studies have established that vascular endothelial growth factor (VEGF) promotes matrix metalloproteinases (MMPs) expression, degrades the extracellular matrix, and mediates HIF‐1α. Thus, HIF‐1α contributes significantly to cell migration, proliferation, and induced EMT in malignant tissue. Several chemotherapeutic drugs have been applied to modulate the HIF‐1α/VEGF signaling pathway, but their hostile outcomes are alarming (Chen et al. 2022). Thus, the application of anthocyanins has been proved effective to modulate these growth factors and associated genetic expressions. The activation of Wnt/β‐catenin signaling is observed in 90% of CRC cases, thus playing a key role in CRC progression. The mechanism is a series of complex steps involving WNT‐protein ligands binding with LRP5/6 membrane receptors, translocation of β‐catenin, and activation of c‐Myc and cyclin D1, subsequently leading to cell proliferation, migration, and oncogenesis (He and Gan 2023). Li, Wang, et al. (2021) reported that anthocyanins (50 μg/mL) and 5‐Fluorouracil and Celecoxib significantly reduced cell proliferation in SW480 and Caco2 CRC cell lines. The combined therapy enhanced *PTEN*, inhibited *EZH2* and *MDR1* expression, and reduced the AKT signaling pathway.

Phosphatidylinositol 3‐kinase (PI3K) is a key factor responsible for cell growth, proliferation, differentiation, and motility and works with mTOR and *AKT* to regulate all these cellular mechanisms. The dysregulation of this PI3K/AKT/mTOR pathway is involved in multiple cancer developments, including breast cancer (BC) (Bertucci et al. 2023). Layosa et al. (2021) studied the anticancer potential of *Prunus avium* ‐derived anthocyanins against MDA‐MB‐453 bc cells and showed that anthocyanin inhibited 50% cell growth via *Akt* and *PLCγ‐1* inactivation and improved caspase‐8 and *Bax/Bcl‐2* expression. Similarly, *Vitis coignetiae Pulliat* isolated anthocyanins (0, 50, 100, 200, and 400 μg/mL) effectively reduced cisplatin‐resistant MCF‐7 cell lines via decreased *p‐NF‐κB, p‐IκB*, and *Akt* signaling. Additionally, they concluded that anthocyanins induced apoptosis by downregulating an anti‐apoptotic protein (XIAP) and enhanced PARP‐1 expression (Paramanantham et al. 2020).

**FIGURE 2:**
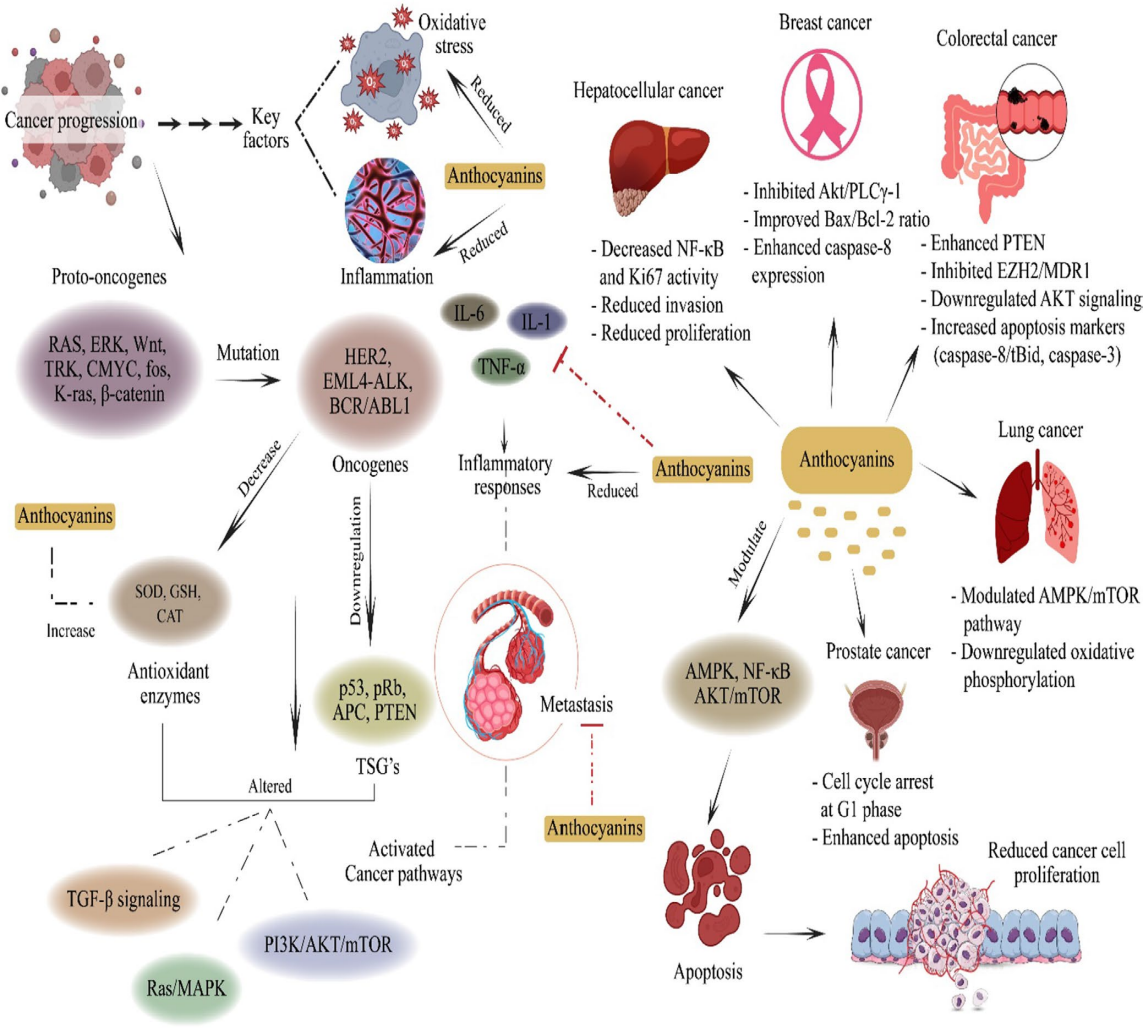
Anti‐inflammatory and anticancer activity of anthocyanins via apoptosis induction, cell proliferation inhibition, cell cycle arrest, modulation of AMPK/mTOR and P13K/AKT pathways, reduction of pro‐inflammatory markers, upregulation of PTEN, PPAR‐γ, and caspase‐8, downregulation of NF‐kB, EZH2, MDR1, and Akt.

Hepatocellular carcinoma (HCC) is one of the most prevalent cancers worldwide and ranks 3rd in terms of cancer mortalities. Kim et al. (2020) reported the anti‐metastatic activity of *Vitis coignetiae Pulliat* extracted anthocyanins against Hep3B cells. The results showed that anthocyanins (100 μg/mL) declined cell invasion and proliferation via inactivation of NF‐κB and Ki67 activity. Luo et al. (2024) investigated the anticancer properties of an anthocyanin‐enriched diet against urethane‐induced lung cancer (LC) in C57BL/6J mice. They found a 0.5% anthocyanins‐rich diet induced apoptosis and inhibited cell proliferation via modulating the AMPK/mTOR signaling pathway and downregulating oxidative phosphorylation in A549 cells. Herrera‐Sotero et al. (2020) worked on blue corn‐derived anthocyanins to determine their anticancer potential against LNCaP prostate cancer cells. They reported reduced cell viability, cell cycle arrest in the G1 phase, and apoptosis. The anti‐inflammatory and anticancer activity of anthocyanins is demonstrated in Figure 2.

### 2.6 Antidiabetic Activity

Diabetes mellitus (DM) is a chronic metabolic endocrine syndrome affecting ~25% of the world population, categorized by raised blood glucose levels either due to inadequate insulin synthesis or impaired body response to insulin. Obesity, smoking, lifestyle and dietary patterns, inherited aberrations, environmental toxins, and chemicals are major risk factors for diabetes. Diabetes can lead to multiple disorders, including hypertension, stroke, inflammatory bowel disorders (IBD), and cancers. Diabetic neuropathy, nephropathy, retinopathy, and diabetic foot are diabetes‐induced microvascular complications that damage other organs of the body, like the heart, kidney, brain, and legs (Zakir et al. 2023). Diabetes can be divided into type 1 diabetes mellitus (T1DM), type 2 diabetes mellitus (T2DM), and gestational diabetes mellitus (GDM). These types differ according to their risk factors and pathophysiology, as T1DM is linked with immune‐mediated β‐cell dysfunction and can be diagnosed by GAD65 autoantibody (Kahaly and Hansen 2016). T2DM involves insulin resistance and reduced insulin sensitivity in muscle, liver, and adipose tissue, leading to hyperinsulinemia, overactivity of pancreatic β‐cells, and eventually β‐cell destruction (Wondmkun 2020). GDM is associated with pregnancy and mainly develops during the 3rd trimester due to hormonal dysregulation, nutrient accumulation, and overweight or obesity (Banday et al. 2020).

The antidiabetic and hypoglycemic effect of medicinal herbs due to rich phytochemistry has been proved in previous studies (Arif et al. 2024). Diabetes can be managed by inhibiting glucose absorption via reduction in certain enzymes, and α‐amylase regulation significantly reduces blood glucose and obesity. In this context, Karcheva‐Bahchevanska et al. (2023) investigated bilberry‐extracted anthocyanins against T2DM and found that M3G and delphinidin‐3‐galactoside were dominant anthocyanins in solvents. They reported that 20.8 μg GAE/mL extract significantly reduced α‐amylase activity. Zhu et al. (2023) reported five blueberry anthocyanins' antidiabetic and hypoglycemic potential in HepG2 cells. They concluded that anthocyanins inhibited α‐glucosidase with IC50 from 68.33 to 218.2 μM. Subsequently, anthocyanins have the potential to lower hyperglycemia and ameliorate diabetes.

Free fatty acid receptor 1 (FFAR1) is identified as a stimulator of glucose‐dependent insulin secretion in β‐cells. It is a molecule stimulated by medium‐to‐long‐chain fatty acids, and activated FFAR1 upsurges calcium ion transportation and insulin synthesis at the cellular level. Moreover, FFAR1 activation triggers the production of the peptide YY, thus leading to a decline in body weight and blood glucose levels (Governa et al. 2021). Anthocyanins from purple corn have been reported to activate FFAR1, promoting insulin production and hepatic glucose uptake. Luna‐Vital and Gonzalez de Mejia (2018) stated that 1 mg/mL anthocyanin‐rich aqueous extract of purple corn improved insulin secretion in INS‐1E cells and enhanced glucose uptake in HepG2 cells. Additionally, 1 mg/mL anthocyanins extract supplementation activated glucokinase (GK), a glucose receptor in the pancreatic β‐cells. The data on clinical trials of anthocyanins on antidiabetic activity and diabetic complications is presented in Table 2.

**TABLE 2:** Clinical trials of anthocyanins on antidiabetic activity and diabetic complications.

Study type	Participants	Dosage/Form	Duration (weeks)	Results	Reference
Randomized controlled trial	59	893 mg	8	**↑**GIP	Christiansen et al. (2023)
Randomized controlled trial	160	320 mg/daily	12	**↑**apoA‐1, apo B, adipsin, **↓**HbA1c, TGs	Yang et al. (2021)
Clinical case‐series study	143	120 mg/daily	4	**↓**TGs, TC, BP, LDL, blood glucose	Tasic et al. (2021)
Randomized controlled trial	20	Half cup blueberries+ EVOO smoothie	2 h before and after intake of smoothie	**↑**Endothelial function	Njike et al. (2021)
Open‐label clinical trial	40	320 mg	4	**↓**FBG, uric acid, IL‐6, IL‐18, TNF‐α	Nikbakht et al. (2021)
Randomized controlled trial	12	Montmorencytart cherry juice, 30 mL/day	1	**↓**FBG, BP, TC, LDL	Desai et al. (2021)
Randomized, double‐blind controlled trial	20	1.4 g/day	4	**↑**glycemic control	Chan et al. (2021)
Randomized crossover clinical trial	35	236.8 mg/day	4	**↑**microbial metabolites, phenyl γ‐ valerolactones, phenolic acids	Zhang et al. (2020)

### 2.7 Anthocyanins and Neurodegenerative Disorders

The nervous system (NS) is a complex system that receives, processes, and responds to sensory information. It comprises the central nervous system (CNS) and peripheral nervous system (PNS). The CNS covers the brain and spinal cord and works with the PNS nerve network. Several neurodegenerative disorders, including Alzheimer's disease, dementia, epilepsy, depression, and anxiety, affect millions of people (Xu et al., 2022). Neurodegenerative disorders (NDs) encompass a diverse group of neurological conditions characterized by a gradual dysfunction of specific neuronal populations. Neuroinflammation is a complex multifactorial process in the CNS, has been notoriously associated with NDs, and fluctuates depending on the disease period. Evidence suggests that neuro‐inflammation, impaired axonal transport (AT), and axonopathy are major pathophysiological factors in NDs (Tesco and Lomoio 2022). Previous studies have proved that AT blockage can damage neuronal homeostasis and contribute to the progression of NDs. Moreover, data have strongly suggested that genetic mutations and disrupted protein expression can dramatically modify the flow and dynamicity of AT. For instance, Tau is a microtubule‐associated protein whose functions are primarily modulated by phosphorylation; thus, tau protein dysfunction and dysregulations directly impact AT dynamics (Combs et al. 2019).

Studies on the neuroprotective role of anthocyanins have proved their ability to reduce neuro‐inflammation, alleviate memory impairment, and improve overall brain function. Concerning this, Ma et al. (2024) investigated the impact of blueberry anthocyanins against arsenic‐induced hippocampal neuron damage. They reported that 200 mg/kg anthocyanins improved antioxidant enzymes and cognitive function and modulated PGC‐1α/NRF2/TFAM expression. The PI3K/AKT signaling pathway is an important pathway mediated by intracellular reactive oxygen species and is involved in cell senescence and apoptosis. Anthocyanins supplementation of Korean black beans (12 mg/kg/day BW) for 30 days proved effective in the mitigation of oxidative stress, neurodegeneration, and improved memory via p‐PI3K/Akt/GSK3β and Nrf2/HO‐1 pathways in APP/PS1 mice. The in vitro model also showed that the supplementation inhibited apoptosis in HT22 cells via suppressing caspase‐3 and PARP‐1 expression and modulating the PI3K/Akt/Nrf2 pathway (Ali et al. 2018). Microglia are the primary immune cells in the CNS, and their inactivation is involved in various NDs. However, anthocyanin and its metabolites have been proven effective in ameliorating neuroinflammation and NDs. Anthocyanin callistephin (100 μM) mitigated LPS‐induced microglial injury in C84B cells via enhanced caspase 3/7 activity, modulated iNOS, TNF‐α, and NF‐κB. Furthermore, it inhibited apoptosis by suppressing *p38* phosphorylation and reduced *BCL‐2* expression (Zhao et al. 2019). Kaewmool et al. (2020) studied the anti‐neuroinflammatory effect of C3G (2.5, 5, 10 μM) against LPS‐induced inflammation in BV2 microglia. They found that pretreatment with C3G substantially inhibited the NO, IL‐1β, IL‐6, and PGE2 production. Moreover, C3G supplementation suppressed iNOS, COX‐2, NF‐κB, and *p38 MAPK* expression, thus attenuating inflammation and preventing apoptosis. Khan et al. (2019) stated that anthocyanins (24 mg/kg/day) for 2 weeks protected against neurodegeneration in LPS‐treated mice. They concluded that 1 week of pretreatment and 1 week of co‐treatment with LPS inhibited ROS production and neurodegeneration while enhancing memory function in mice. The treatment downregulated JNK, thus reduced p‐NF‐kB, IL‐1β, and TNF‐α levels. In addition, neuronal apoptosis was decreased in anthocyanins‐supplemented mice via reduced *Bax*, PARP‐1, cytochrome c, and caspase‐3, while improving the p‐Akt, p‐GSK3β, and Bcl‐2 protein expression.

**FIGURE 3:**
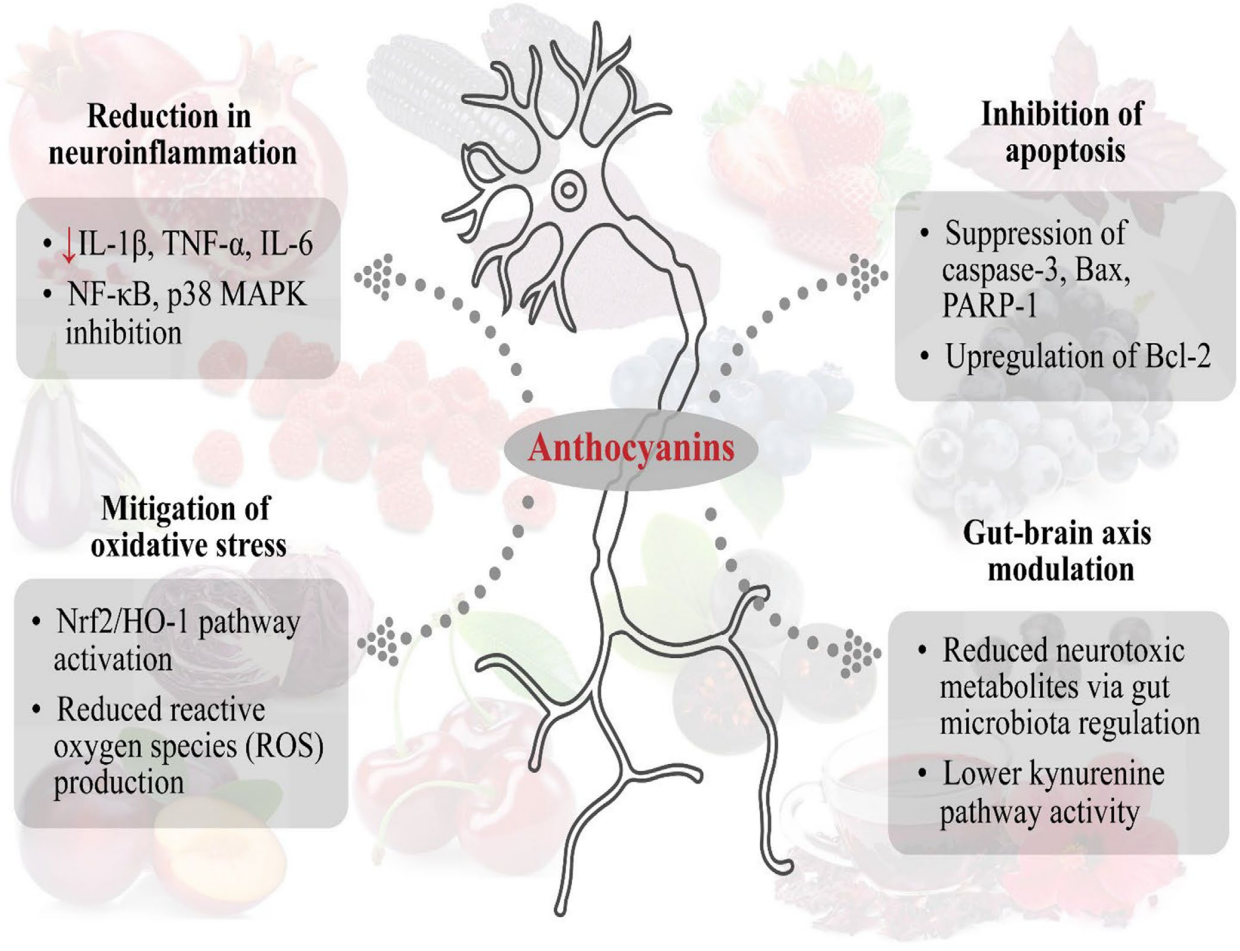
Preventive role of anthocyanins against neuroinflammation and neurodegeneration via regulation of PGC‐1α/NRF2/TFAM, p‐PI3K/Akt/GSK3β, and Nrf2/HO‐1 pathways, upregulation of Bcl‐2, and amelioration of the kynurenine pathway.

The gut‐brain axis (GBA) is a critical factor in neuroinflammation and neurodegeneration because gut dysbiosis, kynurenine pathway impairment, and overproduction of astrocytes could promote neurotoxic metabolites and TGFβ release, which results in the inhabitation of microglia activation and leads to neuronal damage (Kearns 2024). Concerning this, some clinical trials have proved that anthocyanins efficiently improve memory and prevent memory loss in humans. In a randomized, placebo‐controlled trial, blueberry supplementation for 16 weeks improves neural response in older adults with cognitive deterioration (Boespflug et al. 2018). The 72 g of grapes daily for 6 months protected brain metabolism, improved proper superior parietal cortex function, and enhanced working memory in a placebo‐controlled pilot study (Lee et al. 2017). Blueberry concentrate (30 mL) provides 387 mg of anthocyanidins for 12 weeks, which enhances brain perfusion and work capacity and improves cognitive function in healthy elderly adults (Bowtell et al. 2017). Calapai et al. (2017) conducted a randomized, double‐blinded trial for 12 weeks and fed 111 participants with 250 mg/day *V. vinifera* supplement and found that the supplementation improved cognitive function, boosted memory, and alleviated nerve damage. The anti‐neuroinflammatory and neurodegeneration preventive role of anthocyanins is illuminated in Figure 3.

### 2.8 Hepatoprotective Role

The liver is a vital organ that plays a significant role in metabolic processes, including detoxification and eradication of drugs and toxic compounds, which can damage the liver and reduce its function. Fatty liver disease (FLD) is one of the major anomalies, resulting in fat accumulation and dysfunction of the liver. Several inflammatory markers contribute to the disease progression. However, the NLRP3 inflammasome's role in nonalcoholic fatty liver disease (NAFLD) is still unknown. Zhu et al. (2022) reported that anthocyanins (320 mg daily) for 12 weeks ameliorated NLRP3 inflammasome activation and reduced IL‐18, IL‐1β, and caspase‐1 expression in NAFLD patients. Anthocyanins from purple corn effectively reduced liver enzymes in chronic liver injury of mice treated with CCl4. Moreover, anthocyanins reduced MDA levels, downregulated caspase‐3, *Bax*, and cytochrome P450 2E1 protein expression, upregulated *Bcl‐2* expression, and inhibited apoptosis (Cui et al. 2021). Previously, bilberry anthocyanins (200 mg orally) for 7 days attenuated CCl4‐induced hepatotoxicity in rats. Anthocyanin administration inhibited TNF‐α, IL‐6, iNOS, COX‐2, CD68, and lipocalin‐2 and reduced GDH, SDH, MDH, conjugated dienes, TBA, NADPH oxidase, lipid hydroperoxide, H_2_O_2_, and GSSG activity. Anthocyanins also diminished polyamine catabolism and decreased hyperactivation and hyperplasia of Kupffer cells (Popović, Kocić, Katić, Jović, et al. 2019). Table 3 highlights the hepatoprotective role of anthocyanins in animals, humans, and in vitro studies.

**TABLE 3:** Hepatoprotective role of anthocyanins.

Study type	Dose/form/duration	Outcome	Reference
C57BL/6J mice	black rice anthocyanins (14 weeks)	**↓**BW, TGs, TC, steatosis scores, **↑**gut microbiota	Song, Shen, Wang, et al. (2021)
Kunming mice	200, 400 mg/kg	**↓**Ki67, TNF‐α IL‐6, α7nAChR, PI3K‐Akt, Keap1/HO‐1, collagen I	Wei et al. (2024))
C57BL/6J mice	100 and 200 mg/kg	**↓**proliferation of HSCs, modulate circ_0000623/miR‐351‐5p/TFEB	Du et al. (2023)
In vitro, Male rats	25–100 μg/mL, 5 and 10 mg/kg (4 weeks)	**↓**proliferation of HSCs, MDA, HYP, TGFβ1, α‐SMA, **↑**SOD, CAT, GSH‐Px	Zhang, Jiang, et al. (2021)
ICR mice	400 and 800 mg/kg/daily	**↑**SOD, CAT, **↓**MDA, TNF‐α, IL‐17A, IL‐1β, CD4, TGFβR2, p‐Smad2¸ p‐ERK1/2, p‐Smad3, p‐p38, p‐PDGFRβ, p‐AKT, p‐JNK1/2	Dai et al. (2024)
Randomized, Placebo‐Controlled Pilot Trial	74 participants, (320 mg/day), 12 weeks	**↓**ALT, FBG, OGTT, MPO, cytokeratin‐18 M30 fragment	Zhang et al. (2015)
Randomized, placebo‐controlled study	44 subjects, 250 mL bayberry juice, (4 weeks)	**↓**TNF‐α, IL‐8, cytokeratin‐18 fragment M30	Guo et al. (2014)

### 2.9 Nephroprotective Activity

Kidneys are essential due to their remarkable role in metabolic pathways and homeostasis. Moreover, kidneys are responsible for the filtration, reabsorption, and excretion of certain toxic compounds from the body. Multiple factors such as food, toxins, chemicals, pathogens, and OTC drugs can cause renal injury and lead to renal impairment or permanent dysfunction. Anthocyanins have been studied for their role in renal protection, and it has been found that they can significantly protect kidneys from permanent damage. Popović, Kocić, Katić, Zarubica, et al. (2019) highlighted the nephroprotective role of 15 bilberry anthocyanins against CCl4‐induced renal toxicity in rats. They orally supplemented rats with 200 mg anthocyanins for 7 days and concluded that anthocyanins reverse histopathological damage via reverting periglomerular necrosis, dilatation of proximal tubules, and mononuclear infiltrates. Moreover, supplementation reduced NF‐α, NO, MPO, H_2_O_2_, XO, GSSG, β2‐microglobulin, and NGAL and enhanced CAT, SOD, and GPx activity. Similar findings have been reported by Yarijani et al. (2019), who supplemented hepato‐renal toxic rats with (200 or 400 mg/kg) *Malva sylvestris* extract. They concluded that anthocyanins reduced oxidative stress, creatinine, urea, AST, ALT, and ALP levels and inhibited TNF‐α and ICAM‐1 mRNA expression. Bilberry anthocyanins (100 mg/kg/day) exhibited renal protection via reducing MDA, serum creatinine, and AOPP levels in gentamicin‐induced toxic rats (Veljković et al. 2017). *Panax ginseng* anthocyanins mitigated cisplatin‐induced toxicity in rats via ameliorating 4‐hydroxynonenal, MDA, HO‐1, and cytochrome P450 E1 expression. Moreover, alleviation of TNF‐α, IL‐1β, iNOS, and COX‐2 and reduced apoptosis via improving B cell lymphoma 2 and reducing Bcl2‐associated X protein expression was observed (Qi et al. 2017).

Besides animal studies, some clinical trials have been reported, proving anthocyanins potential against renal damage. Spormann et al. (2008) conducted a trial on hemodialytic patients and fed 21 participants 200 mL/day of red fruit juice. A reduction in DNA damage and inhibition of NF‐κB activity and lipid peroxidation was observed. Furthermore, the juice intake improved GSH and SOD levels. In another clinical trial, 32 subjects on hemodialysis were supplemented with 50 mL of red grape juice and 800 IU of vitamin E to study the comparative effect of both treatments on neutrophil NADPH oxidase activity among patients. The study concluded that only grape juice effectively lowered TC and apoB and improved HDL. While both juice and vitamin E supplementation reduced LDL, ICAM‐1, MCP‐1, and neutrophil NADPH oxidase activity (Castilla et al. 2008).

### 2.10 Gut Health and Anthocyanins

The gastrointestinal tract (GIT) plays a crucial role in digestion, absorption, metabolism, and nutrient distribution, making it essential for overall health. However, maintaining a healthy gut has become increasingly challenging due to various factors, including pathogenic infections, irregular eating habits, pesticide residues, toxins, and chemical exposure. These disruptions often lead to gut dysbiosis—an imbalance in the microbial composition of the colon—resulting in inflammation and a cascade of chronic health issues. Therefore, a balanced gut microbiome is critical for sustaining digestive health and preventing related complications (Parkin et al. 2021). Gut dysbiosis is the disruption of the delicate equilibrium between beneficial and harmful bacteria, and this condition occurs due to various factors, such as poor diet, chronic stress, antibiotic overuse, and ailments that affect digestion. The symptoms of gut dysbiosis include bloating, diarrhea, constipation, fatigue, and a compromised immune system (Hrncir 2022). Over time, this imbalance can contribute to more severe health issues, like inflammatory bowel disease (IBD), irritable bowel syndrome (IBS), and even mental health challenges such as anxiety and depression due to imbalanced GBA. Restoring gut health comprises dietary modifications, probiotics, prebiotics, and postbiotics, and addressing underlying triggers to promote a balanced microbiome (Anand et al. 2022).

The stability of anthocyanins and their metabolites in the small intestine is essential in maintaining gut health, as only a limited amount reaches the bloodstream. Once in the gastrointestinal tract, anthocyanins and their metabolites are released, transformed, and fermented by gut microbiota in the large intestine. This process significantly impacts the gut environment, as these compounds modulate the composition of probiotics and promote the production of beneficial short‐chain fatty acids (SCFAs). Through these mechanisms, anthocyanins and their colonic metabolites contribute to improved gut health and overall well‐being (De Vos et al. 2022). The human gut microbiota is categorized into four prominent phyla, that is, *Proteobacteria, Bacteroidetes*, *Actinobacteria*, and *Bacillota*. Furthermore, *Bacillota* and *Actinobacteria* account for ~90% of colonic microbiota (Rinninella et al. 2019). However, despite prime significance, *Lactobacillus* spp. and *Bifidobacterium* spp. are in a small proportion but substantially contribute to gut health. The human gut composition fluctuates in response to interior and exterior stimuli during the host's lifespan. For example, an amplified *Bacillota:Bacteroidetes* ratio is associated with obesity, and a decline in *Lactobacillus* spp. is linked with intestinal diseases (Hou et al. 2022). Therefore, a balance and diversity of probiotics composition are mandatory for gut maintenance and normal functions such as vitamins and SCFAs synthesis, lipid metabolism, and immuno‐modulation (Afzaal et al. 2022).

The combined therapy and encapsulated strategy are more appropriate approaches to improve gut health. In this context, de Aguiar Freire et al. (2024) stated that adding anthocyanins to yogurt can modulate the gut microbiome, improve antioxidant capacity, and reduce inflammation. Additionally, its incorporation could enhance product shelf life, suppress pathogenic microbes, and inhibit enzyme activity. Peng et al. (2020) fed C57BL/6 mice with 200 mg/kg/day anthocyanins extracted from *Lycium ruthenicum* for 12 weeks to determine its impact on gut and overall health. They found that the anthocyanins supplementation improved GSH and GPx while inhibiting ALP, AST, ALT, and MDA levels and also reduced TNF‐α, iNOS, INF‐γ COX‐2, IL‐6, and IL‐1β expression. Moreover, the mRNA expression of Muc1, Occludin, Zo‐1, and Claudin‐1 increased and augmented *Barnesiella, Odoribacter, Eisenbergiella, Coprobacter*, and *Alistipes* microbiota. Morissette et al. (2020) supplemented C57BL/6J obese male mice with 160 mg/kg blueberry powder for 12 weeks to assess its impact on gut health and microbiota. They concluded that supplementation of blueberry powder reduced weight in obese mice, improved insulin sensitivity, and enhanced motor activity. In addition, the supplementation modifies the gut microbiome and increases fecal SCFAs and branched‐chain FA content.

**FIGURE 4:**
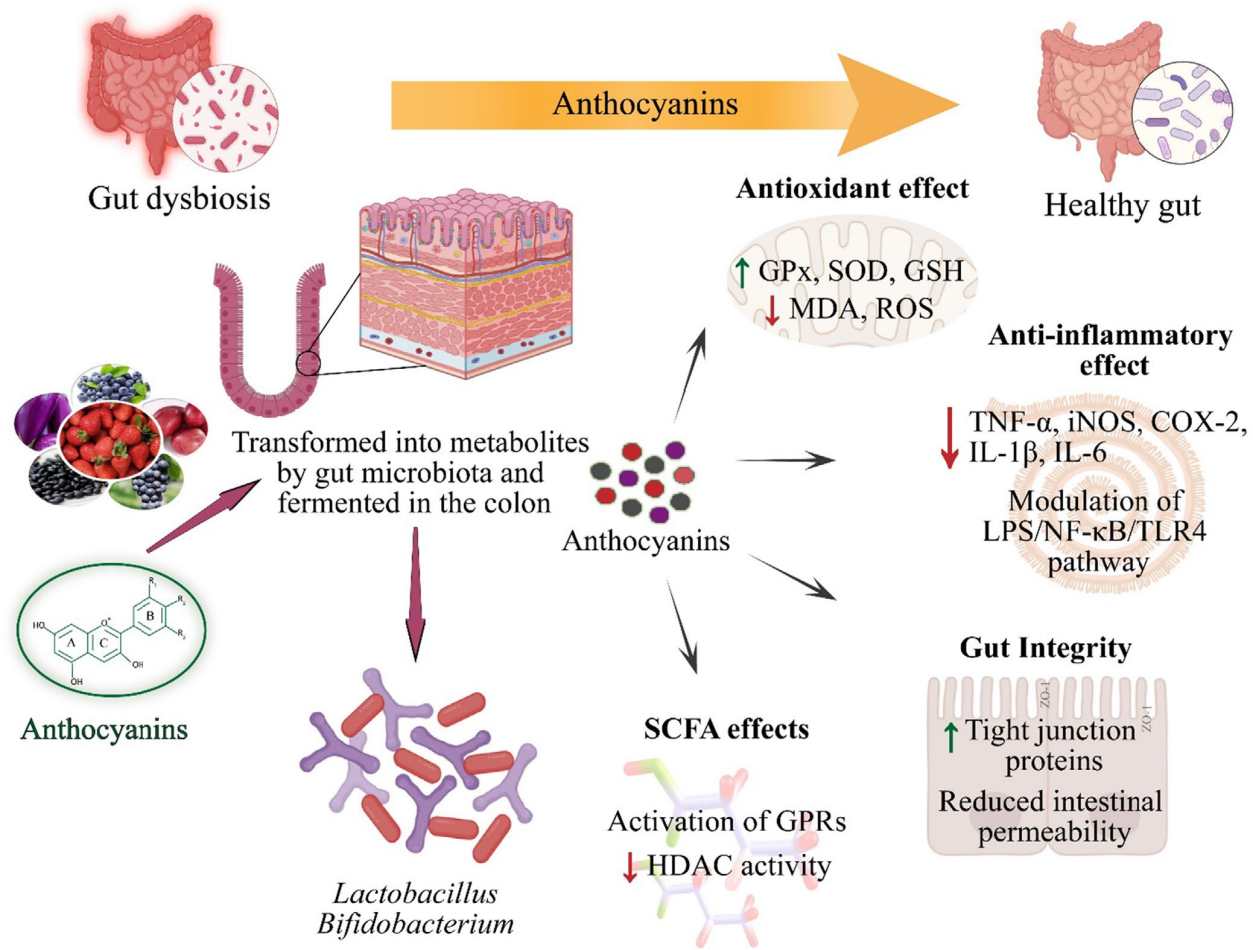
Role of anthocyanins in gut health via regulation of the gut‐brain axis, mitigation of gut dysbiosis, production of SCFAs, enhancement of beneficial gut microbiota, inhibition of INF‐γ, COX‐2, MDA, and IL‐6, and modulation of the NF‐κB/TLR4 pathway.

Malvids anthocyanins are more stable and have been found to improve GPx, SOD, and GSH and reduce MDA levels in the liver of mice. Along with this, malvids anthocyanins modified gut microbiota composition by improving *Firmicutes* and *Lactobacillus* and reducing *Bacteroidetes* (Zheng et al. 2021). Tian, Li, et al. (2021) reported that 50, 100, and 200 mg kg^−1^ anthocyanins for 12 weeks in high‐fat diet‐fed mice ameliorated obesity‐induced inflammation via modulating the LPS/NF‐κB/TLR4 pathway, amplified SCFAs producing bacterial spp. (*Ruminococcaceae*, *Akkermansia*, and *Muribaculaceae*), while inhibiting *Helicobacter* spp. and lipopolysaccharide levels. Moreover, SCFAs activated GPRs and reduced HDAC and intestinal permeability. Mo et al. (2022) investigated mulberry anthocyanins (MAS) against DSS‐induced ulcerative colitis in C57BL/6J mice. They supplemented mice with 200 mg/kg BW MAS for 14 days and found that MAS supplementation attenuated oxidative stress, inflammation, and colon tissue damage. The MAS recovered ZO‐1, claudin‐3, and Occludin expression and reduced dysbiosis via enhancing *Akkermansia*, *Allobaculum*, and *Muribaculaceae* and inhibiting *Escherichia‐Shigella*. The impact of anthocyanins on gut health is highlighted in Figure 4.

### 2.11 Immuno‐Modulatory Impact

The protection against pathogenic invasion and inflammatory assaults is called the immune system, a complex chain of different cells and proteins working together through signaling mediators. The human immune system covers three types: innate (first line of defense), complementary (cascade of proteins), and acquired (second line of defense). The innate immune system attacks and eradicates pathogens; complementary proteins work as bridges or signaling chemicals between the innate and acquired immune systems, and acquired immunity comprises B and T lymphocytes involved in memory, tagging, and killing of antigens. This protective complex system, if somehow compromised, causes subsequent impaired immunity, pathogenic invasion, and even death (Munteanu and Schwartz 2022). Anthocyanins have been reported to modulate the immune system, as Tan et al. (2023) studied the impact of blueberry anthocyanins against fluoride‐induced immune injury in Wistar rats. They fed rats with 100 mg/kg BW anthocyanins and found that anthocyanin supplementation alleviated immune cell apoptosis, inhibited OS, reduced pro‐inflammatory markers, and modulated immunoglobulins.

Pedret et al. (2024) recently reported immuno‐modulation in hypercholesterolemic subjects fed on 80 g day^−1^ red apples enriched in anthocyanins. The randomized trial concluded that the consumption of apples for 6 weeks inhibited C‐reactive protein, IL‐6, lipid profile, and P‐selectin. Moreover, C1q, C4, and Factor B were also reduced in subjects. *Melastoma malabathricum* fruit 5% (total anthocyanins) was fed to Banana shrimp in the diet for 1 month to study its impact on the immune system. The results showed that HCs, PO, CAT, GPXs, and SOD significantly improved in shrimps (Onsanit et al. 2020). Anthocyanins, delphinidin‐3‐O‐glucoside, and C3G (100–600 μg/mL) were evaluated against HCT‐116 and HT‐29 CRC cell lines. The 50% inhibition concentrations for HCT‐116 and HT‐29 were 396 ± 23 and 329 ± 17 (μg/mL) respectively. The molecular docking demonstrated that C3G exhibited the highest potential to inhibit immune checkpoints (PD‐1 and PD‐L1). The findings verified that C3G and D3G derivatives tend to bind and inhibit immune checkpoints (Mazewski et al. 2019).

### 2.12 Hypolipidemic and Cardioprotective Role

The rapid lifestyle change has upset human life in several ways and led to severe metabolic diseases such as hyperlipidemia. Hyperlipidemia is a condition that includes numerous genetic and acquired ailments that describe augmented lipid levels within the human body. It can also be defined as the HDL levels < 10th percentile and LDL, total cholesterol, and triglycerides levels > 90th percentile (Miao et al. 2020). The hyperlipidemic state is linked with multiple complications, including stroke, cardiac arrest, and coronary artery disease. The association between hyperlipidemia and cardiovascular disorders (CVDs) has been discussed because high lipid levels are involved in plaque formation and atherosclerosis, thus leading to cardiac problems (Hussain et al. 2024). According to WHO, CVD accounted for ~17.9 million people's deaths in 2019, representing ~32% of all global expiries, and ~85% were due to heart failure and stroke, therefore making them a leading cause of death worldwide. The significant risk factors for CVDs include unhealthy diet (sodium, cholesterol, saturated & trans fats), low physical activity, and tobacco and alcohol consumption. Moreover, some other health anomalies, such as hypertension (HTN), diabetes, obesity, renal abnormalities, and disturbed mental health, can also contribute to CVD development (Teo and Rafiq 2021).

Atherosclerosis is considered the leading cause of CVDs, characterized by thickening and hardening of the arterial walls, and plasma cholesterol level > 150 mg/dL is a leading cause of atherosclerosis development. Coronary artery disease (CAD) and arterial hypertension (AH) are other major contributors to cardiovascular disorders. CAD is caused by plaque formation within the intima of the blood vessels, resulting in inflammation and inadequate supply of blood, nutrients, and oxygen to cardiomyocytes. Consequently, this leads to rupture of the endothelial lining, thrombosis, limb ischemia, myocardial infarction, stroke, and even death (Bauersachs and Zannad 2018). The AH could be one of the most lethal risk factors due to very few symptoms and could even cause other chronic issues like renal dysfunction. It is characterized by ≥ 140 mmHg systolic blood pressure and ≥ 90 mmHg diastolic blood pressure (Wang and Khalil 2018). Several mechanisms are involved in the development and progression of atherosclerosis and CVDs. The dysfunction of the endothelium, oxidizing factors such as IL‐1, IL‐6, TNF‐α production, DNA methylation via DNMT1, and genetic mutations in *DNMT3A*, *JAK2*, *ASXL1*, and *TET2* are significant events in cardiovascular disorders progression (Du et al. 2021).

**FIGURE 5:**
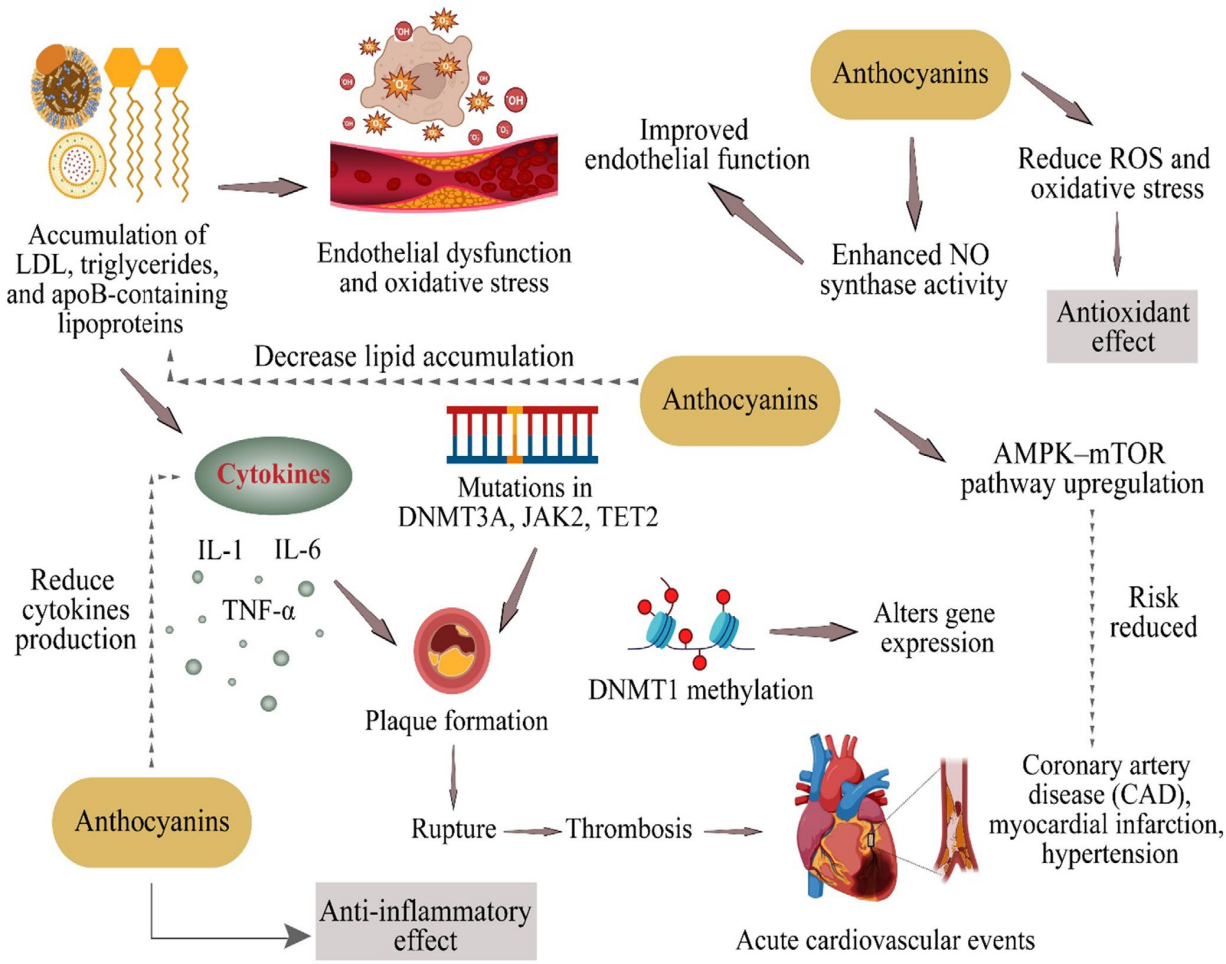
Hypolipidemic and cardioprotective role of anthocyanins via reducing LDL, VLDL, TGs, and TC, improved GBA, upregulation of ATP6 V0C, ZO‐1, and ATG4D expression, improved endothelial function, and decreased plaque formation.

Studies have proved that the deficiency of apoE develops hypercholesterolemia and atherosclerosis in murine models. In addition, LDL and apoB‐containing lipoproteins accumulation in large and medium arteries also promotes atherosclerosis (Oppi et al. 2019). Cyanidin‐3‐O‐β‐glucoside (C3G) (0.2%) in the AIN‐93 diet significantly improves endothelial NO synthase phosphorylation, increases endothelial repair, and reduces atherogenesis in apoE−/− mice (Zhang et al. 2013). Di Pietro et al. (2022) reported that combined omega‐3‐lysine complex and C3G reduced oxidative stress and inhibited vascular dysfunction. The anti‐aging potential of anthocyanins could be another possible outcome to protect the cardiovascular system because the senescence of vascular endothelial cells leads to aging‐related vascular anomalies. Anthocyanin‐ *Aronia melanocarpa* extract (1–25 μg/mL) protected endothelial progenitor cells by reducing ROS production and improving telomerase activity (Parzonko et al. 2015). Li et al. (2019) found that bilberry anthocyanin supplementation (20 mg/kg BW/day) in aging female rats improved CAT, SOD, and HDL and reduced LDL, MDA, TC, TGs, and VLDL. Furthermore, the supplementation upregulated *OCLN, ATP6 V0C, ATG4D, CTSB*, and *ZO‐1* expression, promoting AMPK–mTOR‐induced autophagy. Wang et al. (2020) concluded that C3G (50, 100 mg/kg BW) reduced blood lipid levels, recovered artery wall structure, inhibited inflammatory cytokines, upregulated miR‐204‐5p, and down‐regulated *SIRT1* expression. The anthocyanins‐rich extract of red Chinese cabbage (10, 150, 300 mg/kg) attenuated aortic inflammation, reduced plaque formation, and inhibited cytokines production in ApoE−/−, C57BL/6J mice (Joo et al. 2018). Despite strong evidence of anthocyanins' cardiovascular protective effect, a few studies contradict and support the theory of no significant impact of anthocyanins against CVDs. Regarding this, Aboufarrag et al. (2022) reported that 320 mg anthocyanins capsules extracted from bilberry for 28days showed no effect on LDL, TGs, TC, paraoxonase‐1, and ApoB in a randomized, placebo‐controlled trial. They proposed that other clinical/human trials proved the protective effects of anthocyanins due to long‐term consumption. Therefore, short‐term use did not show any remarkable impact. The hypolipidemic and cardioprotective role of anthocyanins is displayed in Figure 5.

### 2.13 Antibacterial Properties

Multiple studies have highlighted the antibacterial properties of anthocyanins against a wide range of pathogenic microbes. The primary mechanism of action against microbes is the destruction of cell walls and membranes. Moreover, disruption in homeostasis could also lead to microbial inactivation/degradation. Deng et al. (2021) investigated *A. melanocarpa* anthocyanins against *E. coli* , and they found that the MIC and MBC of anthocyanins against *E. coli* were 0.625 mg/mL and 1.25 mg/mL. The study concluded that anthocyanins damage the cell wall and cell membrane, along with DNA disruptions of bacteria. The chitosan, polyvinyl alcohol, and nano‐ZnO, comprising purple potato anthocyanins biofilms, were developed to determine their antibacterial potential against *E. coli* and *S. aureus* . The inhibition zone for *S. aureus* and *E. coli* was 15.09 ± 0.32 mm and 11.72 ± 1.47 mm. Moreover, the moisture content of films was reduced, whereas mechanical resistance significantly increased (Liu et al. 2021). Recently, Liu et al. (2024) studied the antibacterial properties of black bean skin anthocyanins against *V. parahaemolyticus* , and it was found that the anthocyanins altered cell membrane integrity and interfered with cell membrane proteins, thus leading to bacterial inhibition. The MIC value was 10 μg/mL, and the inhibition rate reached 91.94% after exposure to 1 MIC. The ZIF‐8 NPs‐based film, containing blueberry anthocyanins, hinokitiol, and sodium alginate matrix, was developed to determine its antibacterial properties against *E. coli* and *S. aureus* . The results showed that the ZIF‐8‐BA‐HIN‐SA‐based film showed the highest antibacterial activity (96.23%), and the film enhanced the shelf life of pork to 6 days at 4°C. The E‐nose evaluation disclosed that the film inhibits unpleasant, irritating odors‐producing components (Guo et al. 2024).

The anthocyanins extracted from *P. granatum* peel, *C. annuum* fruit, and *Bougainvillea spectabilis* flowers showed great antibacterial activity against *S. aureus* , *Streptococcus pyogenes* , *L. monocytogenes* , *L. ivanovii* , *K. oxytoca* , *Salmonella typhimurium* , *P. aeruginosa* , and *E. coli* . The *P. granatum* peel showed the highest antibacterial properties, followed by *C. annuum* fruit and *Bougainvillea spectabilis* flowers (Abdelrahman et al. 2024). Colorimetric films are emerging as an effective indicator of freshness to meet the diverse demands of consumers. Qi et al. (2022) developed a nisin and anthocyanins‐based colorimetric film, and it showed high antioxidant and antibacterial activity against *S. aureus* , improving shelf life and freshness of shrimps at 4°C. The anthocyanins extracted from *Amelanchier alnifolia* via XAD‐8 macroporous resin exhibited strong antibacterial activity against *S. aureus* , *E. coli* , and *Bacillus subtilis* . Moreover, the UPLC‐MS analysis revealed that C3G, delphinidin‐3‐glucoside, and malvidin‐3‐glucoside are dominant anthocyanins (Zhang, Cui, et al. 2021).

### 2.14 Miscellaneous

Studies have proved that anthocyanins protect against intestinal disorders through gut microbiota modulation. Li, Zhu, and Zeng (2021) reported that 120 g/BW anthocyanin‐rich potato consumption alleviated IL‐6, IL‐17, and IL1‐β expression, shortening of colon length, mucin 2 levels, and MPO activity in DSS‐induced colitis C57BL/6 mice. Peng et al. (2019) mentioned the anti‐inflammatory and gut‐modulation role of *Lycium ruthenicum* anthocyanins against DSS‐induced colitis in 57BL/6 mice. They concluded that anthocyanins and P3G compounds attenuated IL‐6, IL‐1β, IFN‐γ, and TNF‐α expression and enhanced ZO‐1 and claudin‐1. Furthermore, a reduction was noticed in *Helicobacter* spp., *Oscillibacter* spp., *Lachnospiraceae* spp., and *Parabacteroides* spp., which are associated with colitis. Anthocyanins and anthocyanin‐rich foods have been found to reduce osteoarthritic inflammation via inhibiting MAPK and NF‐kB pathways (Panchal et al. 2022). Anthocyanins have been linked with mitigating retinal inflammation during viral infections (Ahmad et al. 2015). Bilberry anthocyanin‐rich extract (500 mg/kg BW) reduced LPS‐induced retinal inflammation and protected vision in C57BL/6 mice. Moreover, the extract inhibited STAT3 activation and IL‐6 expression in infected mice (Miyake et al. 2012). Bilberry anthocyanins inhibited photo‐oxidation of A2E, a pigment that can cause light‐induced damage to the cell upon accumulation in retinal epithelial cells (Shim et al. 2012). *Lycium ruthenicum* anthocyanin extract (500 mg/kg/d) efficiently attenuated testicular damage and prevented spermatogenesis in cadmium‐induced testicular toxicity via upregulating the SIRT1/Nrf2/Keap1 pathway. In addition, supplementation improved testosterone and inhibin B levels, enhanced SOD and CAT activity in ICR mice (Dong et al. 2024).

### 2.15 Safety Recommendations and Toxicity

The safety of bioactive compounds is a primary concern before their application because over and long‐term consumption is associated with several health risks, including genotoxicity, cytotoxicity, biochemical alterations, and organ dysfunction. The evidence‐based reports lack safety regarding compounds. Regarding anthocyanins, the joint FAO/WHO committee on food additives has recommended an acceptable daily intake of 2.5 mg/kg/day for grape‐skin extracts anthocyanins, but this recommended dose is not for general anthocyanins. China is the first country to establish a recommended intake of anthocyanins (50 mg/day), but it has still not issued a Tolerable Upper Intake Level (TUIL) (Chinese Nutrition Society 2013). Animal‐based studies have proved its safety with no toxic events at these levels: rats (20 mg/kg/day), mice (25 mg/kg/day), guinea pigs (> 3 g/d for 15 or 90 days), and beagle dogs (> 2.4% BW) (Wallace and Giusti 2015). However, the European Food Safety Authority decided the recently available toxicologic database could not confirm a numerically suitable daily intake for anthocyanins. Regarding the safety of anthocyanins, Abdelgadir et al. (2024) supplemented Wistar rats with different doses of anthocyanins (10 mg/kg BW, orally), anthocyanins and curcumin (10 + 10 mg/kg BW), and anthocyanins and sodium nitrite (10 + 5 mg/kg BW) per day for repeated sub‐acute toxicity (28 days). They found augmented AST, CK, and LDH enzyme activity, altered hematological parameters, and histopathological abnormalities in the liver and kidneys. They concluded that anthocyanins combined with other substances might induce toxic effects. Shuping et al. (2023) conducted acute and sub‐acute toxicity studies on Sprague Dawley rats and mice. For acute oral toxicity, mice were fed on 8000 mg/kg/day *Lycium ruthenicum* anthocyanins extract via intragastric route, and no toxic sign was observed in mice during 14 days. For sub‐acute toxicity, rats were administrated (intragastric) with extract for 30 days, and no toxicity was recorded; however, monocytes increased, and AST/ALT ratios declined in rats. The study concluded that there was no toxic effect at this dose; however, minor changes were observed in hematological and biochemical indices.

### 2.16 Industrial Applications of Anthocyanins

Anthocyanins have many applications in the food, cosmetics, and pharmaceutics industries. Mainly, they are used as food colorants due to their prominent colors and photochromic features. However, recent strategies have been developed due to their unstable nature to make them more stable and have a longer shelf life. pH is one of the most critical factors that can affect anthocyanins' color stability, and they exhibit bright red to purple and bright blue depending on the pH conditions. It has been observed that the anthocyanins produce purple and blue colors at basic pH, but at this pH, they begin to degrade. Thus, the main limitation of anthocyanin applications in the food sector is that most foods are moderately acidic to moderately basic, subsequently resulting in anthocyanin loss (Câmara et al. 2022).

The nanoencapsulation is more suitable for maintaining anthocyanins' color and structure. Machado et al. (2022) used microencapsulated red cabbage anthocyanin‐rich extract as a food colorant. The study proved that maltodextrin and Arabic gum improved the thermal stability of microparticles. Moreover, the 25:25 maltodextrin: Arabic gum was ideal, and nanoparticles were stable at > 40°C. The effect of grape pectic polysaccharides on mv3Glc thermal stability was evaluated, and it was found that polysaccharides substantially improved anthocyanins' thermal stability. They concluded that fewer branched structures and pectin flexibility enhance mv3Glc thermo‐stabilization (Fernandes et al. 2020). In addition, co‐pigmentation and nanoliposome approaches have been applied to improve stability. Ghareaghajlou et al. (2022) developed phosphatidylcholine‐based nanoliposomes of red cabbage anthocyanins. They found that during 21 days of storage, anthocyanin‐nanoliposomes were more intact at 4°C than at 25°C and can be used as functional food formulation in non‐water‐soluble mediums.

The increased global demand for cosmetics products such as nail paints, skin & hair care items, deodorants, and hair dyes has upsurged production. The recent trends have shifted toward natural origins, and consumers have been demanding safe cosmetic products with no adverse impact. Therefore, anthocyanins are extensively used in the cosmetic industry due to their eye‐catching colors. Blackcurrant fruit waste anthocyanins were used as hair dyes (Rose et al. 2018). Clay minerals and oil emulsions with anthocyanins are suitable methods to enhance anthocyanins' stability in skin care products. *Malus dosmestica* peel anthocyanins were used to develop an oil/water emulsion, and it was noticed that 3% anthocyanin emulsion was stable during 90 days of storage (Khan et al. 2021). Anthocyanins in sunscreen are being used to maintain healthy skin and melanin content. Purple sweet potato extracts (0.61 mg/100 g cream) concentration absorbed 46% of UV radiation (Correia et al. 2021). Furthermore, the Nivea and L'Oreal groups use anthocyanins from different sources in their commercial products.

### 2.17 Nutraceutical Applications

Nutraceuticals, with their health‐promoting attributes, are used worldwide. Nutraceuticals are nutrients that can regulate physiological and biochemical mechanisms, thus having advantageous health results. These outcomes can be accomplished by pharmaceutical supplements or by the amalgamation of extracts rich in bioactive compounds in processed foods. Several commercial supplements have been developed from anthocyanins and sold under different brand names since the 2000s. The Scandinavian bilberries and black currants anthocyanins extract are sold under Medox, and this 80 mg supplement is used for CVD patients, and several researches have proved its effectiveness (Tian, Zhao, et al. 2021). Bilberry extract, sold under the name of Mirtoselect, contains 36% of anthocyanins extract, and it has been reported that 160–320 mg/day of bilberry extract could improve eye health and memory and can reduce CVD risk (Riva et al. 2017). Mirtogenol, a combination of Mirtoselect and Pycnogenol (French maritime pine bark extract), is used to manage intraocular hypertension. Mirtogenol tablets (twice/day) for 6 months decreased intraocular hypertension (26 mmHG to 22 mmHG) in 20 subjects (Steigerwalt Jr et al. 2008). Besides supplements, Koka, a Singapore brand, used purple wheat to produce noodles pasta. Moreover, black wheat can reduce obesity and obesity‐associated metabolic disorders. Fortified black, blue, and purple wheat with zinc can inhibit zinc deficiency and malnutrition, and these wheat varieties showed more excellent thermal stability and lesser amino acid cooking losses (Sharma et al. 2022).

### 2.18 Limitations and Future Perspectives

Despite the well‐documented health benefits of anthocyanins, there are some limitations that must be addressed to fully harness their potential. One of the main challenges is their low bioavailability. After consumption, anthocyanins undergo rapid metabolism and degradation in the GIT, leading to reduced absorption and limited systemic circulation. This makes it difficult to achieve optimal therapeutic effects from dietary sources alone. Another limitation is the variability in anthocyanin content among different food sources. Factors such as plant variety, cultivation conditions, and food processing methods can significantly influence anthocyanin levels. Furthermore, anthocyanins are highly unstable, and their degradation can be accelerated by exposure to heat, light, and pH changes, further complicating their application in food and pharmaceutical industries. The lack of standardized dosing guidelines is another challenge. Although several studies highlight the health‐promoting effects of anthocyanins, there is no harmony on the required daily intake for optimal benefits. Additionally, most research is based on in vitro and animal models, with limited large‐scale human clinical trials to validate their long‐term safety and efficacy.

To overcome these restrictions, future research should focus on improving anthocyanin bioavailability through advanced delivery systems such as nanoparticles, liposomes, and microencapsulation. Genetic engineering and selective breeding of plants could also enhance anthocyanin content and stability. More clinical trials are required to establish clear dosage recommendations and understand anthocyanins' long‐term effects in human populations. Moreover, the development of functional foods and supplements with optimized anthocyanin formulations may offer more effective health benefits. With continued research and innovation, anthocyanins have the potential to become a keystone of personalized nutrition and preventive healthcare.

## 3 Conclusion

Anthocyanins are natural coloring compounds in purple, red, blue, orange, and black fruits, vegetables, and flowers. Several research studies highlight their role in reducing the risk of chronic diseases, including CVD, type 2 diabetes, obesity, and neurodegenerative disorders. Their ability to modulate OS, improve endothelial function, and regulate glucose metabolism makes them valuable functional nutrients. Additionally, anthocyanins exhibit anti‐cancer effects by inhibiting tumor growth and promoting apoptosis in cancer cells. Their neuroprotective properties may help prevent cognitive decline and Alzheimer's disease. Regular dietary intake of anthocyanin‐rich foods, such as berries, red cabbage, and black rice, can contribute to overall health and longevity. However, there is a need to develop more innovative strategies to enhance their stability, and clinical trials need to be conducted to explore their bioactivities via possible mechanisms.

## Author Contributions

**Ahmad Mujtaba Noman:** conceptualization (equal), writing – original draft (equal). **Muhammad Tauseef Sultan:** conceptualization (equal), resources (equal), writing – original draft (equal). **Muhammad Maaz:** investigation (equal), writing – review and editing (equal). **Aimen Mazhar:** conceptualization (equal), writing – review and editing (equal). **Naima Tariq:** writing – review and editing (equal). **Muhammad Imran:** resources (equal), writing – original draft (equal). **Muzzamal Hussain:** data curation (equal), supervision (equal), validation (equal), visualization (equal). **Ahmed Mujtaba:** data curation (equal), investigation (equal), methodology (equal). **Mohamed A. Abdelgawad:** resources (equal), validation (equal), visualization (equal). **Ehab M. Mostafa:** writing – review and editing (equal). **Mohammed M. Ghoneim:** investigation (equal), writing – original draft (equal). **Samy Selim:** conceptualization (equal), data curation (equal). **Entessar Al Jbawi:** data curation (equal), supervision (equal).

## Conflicts of Interest

The authors declare no conflicts of interest.

## Data Availability

The data that support the findings of this study are available on request from the corresponding author.
